# Comparison of Fat-Free Mass and Ideal Body Weight Scalar for Anesthetic Induction Dose of Propofol in Patients with Morbid Obesity: A Double-Blind, Randomized Clinical Trial

**DOI:** 10.5812/aapm-140027

**Published:** 2023-12-13

**Authors:** Soudabeh Djalalimotlagh, Mahmoud Reza Mohaghegh, Mohammad Reza Ghodraty, Amineh Shafeinia, Faranak Rokhtabnak, Tina Alinia, Farnoosh Tavakoli

**Affiliations:** 1 Department of Anesthesiology, Firoozgar Hospital, School of Medicine, Iran University of Medical Sciences, Tehran, Iran; 2 Department of Anesthesiology, Hashemi Nezhad Hospital, School of Medicine, Iran University of Medical Sciences, Tehran, Iran; 3 Department of Anesthesiology, Akbar Abadi Hospital, School of Medicine, Iran University of Medical Sciences, Tehran, Iran; 4 Department of Emergency, Shohada Salamat Hospital, School of Medicine, Iran University of Medical Sciences, Malard, Iran; 5 Department of Anesthesiology, School of Medicine, Iran University of Medical Sciences, Tehran, Iran

**Keywords:** Bariatric Surgery, Body Composition, Fat-Free Mass, Ideal Body Weight, Morbid Obesity, Propofol

## Abstract

**Background:**

Obesity is a growing problem worldwide and can affect both the pharmacodynamics and pharmacokinetics of various drugs, including anesthetics, resulting in the under-or overdosing of certain drugs. There is no consensus on the ideal dosing regimen for obese populations.

**Objectives:**

In this study, 2 weight-based dosing of propofol used for induction of anesthesia were compared in terms of the onset of action time, adequacy of anesthesia, and effects on hemodynamic indices (eg, heart rate [HR] and blood pressure).

**Methods:**

In this randomized, double-blind clinical trial, 40 patients with morbid obesity (MO) scheduled for bariatric surgery with body mass index (BMI) > 35, age 18 - 59 years, American Society of Anesthesiologists physical status (ASA-PS) II and III were randomly divided into 2 groups, using block randomization method, to receive 2 mg/kg of propofol for induction of anesthesia based on either fat-free mass (FFM) group or ideal body weight (IBW) group. The primary outcome was the time duration to reach the bispectral index (BIS) ≤ 60. Time to the disappearance of eyelash reflex, signs of inadequate anesthesia (ie, BIS > 60, straining during intubation, or eye-opening), requirements for additional doses, and hemodynamic indices (including HR and mean arterial pressure [MAP]) were also compared.

**Results:**

The mean time to reach BIS ≤ 60 was 134.1 s in the FFM group and 148.7 s in the IBW group. This difference was not statistically significant (P = 0.334). The time of disappearance of eyelash reflex was also not significantly different between the study groups (P = 0.814). However, 2 patients in the FFM group and 8 patients in the IBW group showed signs of inadequate anesthesia and required additional doses. This difference was statistically significant (P = 0.032). Hemodynamic variables, before and 2 min after propofol induction dose administration were comparable between the study groups (P = 0.520, P = 0.327, P = 0.847, P = 0.516 for pre-intervention MAP, post-intervention MAP, pre-intervention HR, and post-intervention HR, respectively).

**Conclusions:**

Propofol dosing, based on FFM and IBW, for induction of anesthesia, provides comparable onset time of action and hemodynamic effects; however, in terms of the adequacy of anesthesia, the dosing based on FFM is more favorable compared to the dosing based on IBW.

## 1. Background

Obesity is one of the major health problems worldwide, and its prevalence is increasing in developed and developing countries (1-3). It is a major risk factor for many diseases and is associated with conditions such as type 2 diabetes, cardiovascular diseases (such as hypertension), stroke, obstructive sleep apnea (OSA), and hyperlipidemia, and many cancers. Therefore, it imposes a financial burden on the health care system and also increases mortality (4-7). Due to the increasing prevalence of morbid obesity (MO), the number of surgeries performed on this population has increased accordingly, including general, cosmetic, and bariatric surgeries (8). Anesthesiologists may face many challenges in treating these patients through various stages of surgery and postoperative care. Obesity alters the pharmacodynamics (PD) and pharmacokinetics (PK) of various drugs (9). The physiological and anthropometric changes observed in obesity affect the PK properties of most drugs. Although fat mass and lean body weight (LBW) are increased with obesity, there is a marked increase in the fat percentage of body weight in MO. These differences in the proportion of body composition affect the volume of distribution of some drugs in patients with MO. Increases in cardiac output and total blood volume, as well as regional changes in blood flow (which occur with MO), can also affect peak plasma concentrations, clearance, and elimination half-life of many anesthetics (10). Morbid obesity can also alter the PD properties of some drugs. Disturbances in metabolic, cardiac, and respiratory function may increase anesthetic side effects, narrow the therapeutic window, and increase the risk of anesthesia in this population (11-13). Increased pharynx and chest wall fat composition and increased incidence of OSA increase the risk of respiratory adverse events after anesthesia and alter the PD profile of anesthetic agents (11). For this reason, an individualized dosing scalar that takes into account the changes in body composition should be used for patients with MO, especially in anesthesia (ie, anesthetics, neuromuscular blockers, and opioids) and critical care settings (14-17).

Propofol is a highly lipophilic sedative-hypnotic drug that rapidly distributes from blood to tissues and depresses the central nervous system through positive regulation of gamma-aminobutyric acid and inhibition of N-methyl-D-aspartate (18, 19). It is a widely used intravenous (IV) anesthetic due to its rapid onset, short duration of action, and ease of titration for anesthesia induction (20). The different dosing scalar of propofol for induction of anesthesia has been recommended, including dosing based on total body weight (TBW) (21), LBW (22, 23), corrected body weight (ie, LBW + 60% [TBW-LBW]) (24), and ideal body weight (IBW) (25). However, it has been shown that TBW-based medication in morbidly obese individuals can result in higher plasma concentrations, overdose, and adverse outcomes (26). Several studies have shown that the required dose for induction of anesthesia was significantly reduced when using LBW (23, 24).

## 2. Objectives

This study was designed to find an appropriate dose for induction of anesthesia in patients with MO, providing enough depth of anesthesia while having a minimal effect on hemodynamic variables. Therefore, we proposed that propofol dosing for induction of anesthesia, based on fat-free mass (FFM), provides a more favorable depth of anesthesia, although it does not have a robust effect on hemodynamic variables.

## 3. Methods

### 3.1. Study Design and Participants

The study protocol of this double-blind, randomized clinical trial was approved by the Ethics Committee of Iran University of Medical Sciences (IR.IUMS.FMD.REC.1399.431) and registered in the Iranian Registry of Clinical Trials (IRCT20201024049135N1). All steps of the study were conducted following the Declaration of Helsinki. Forty patients with MO, body mass index (BMI) > 35, aged 18 - 59 years, the American Society of Anesthesiologists physical status (ASA-PS) II and III, undergoing bariatric surgery at Firoozgar Hospital from April 2021 to September 2022 were included in the study. Written informed consent was obtained from the patients. Exclusion criteria included significant systemic disease, hepatic or renal dysfunction, predicted or known difficult airway, need for awake intubation, history of allergy to the study drugs, behavioral disorders, use of psychiatric medications, and history of drug abuse. The patients were randomly divided into 2 groups using the stratified block randomization method. They received an induction dose of 2 mg/kg of propofol for anesthesia based on either the FFM group or the IBW group.

**Figure 1.**
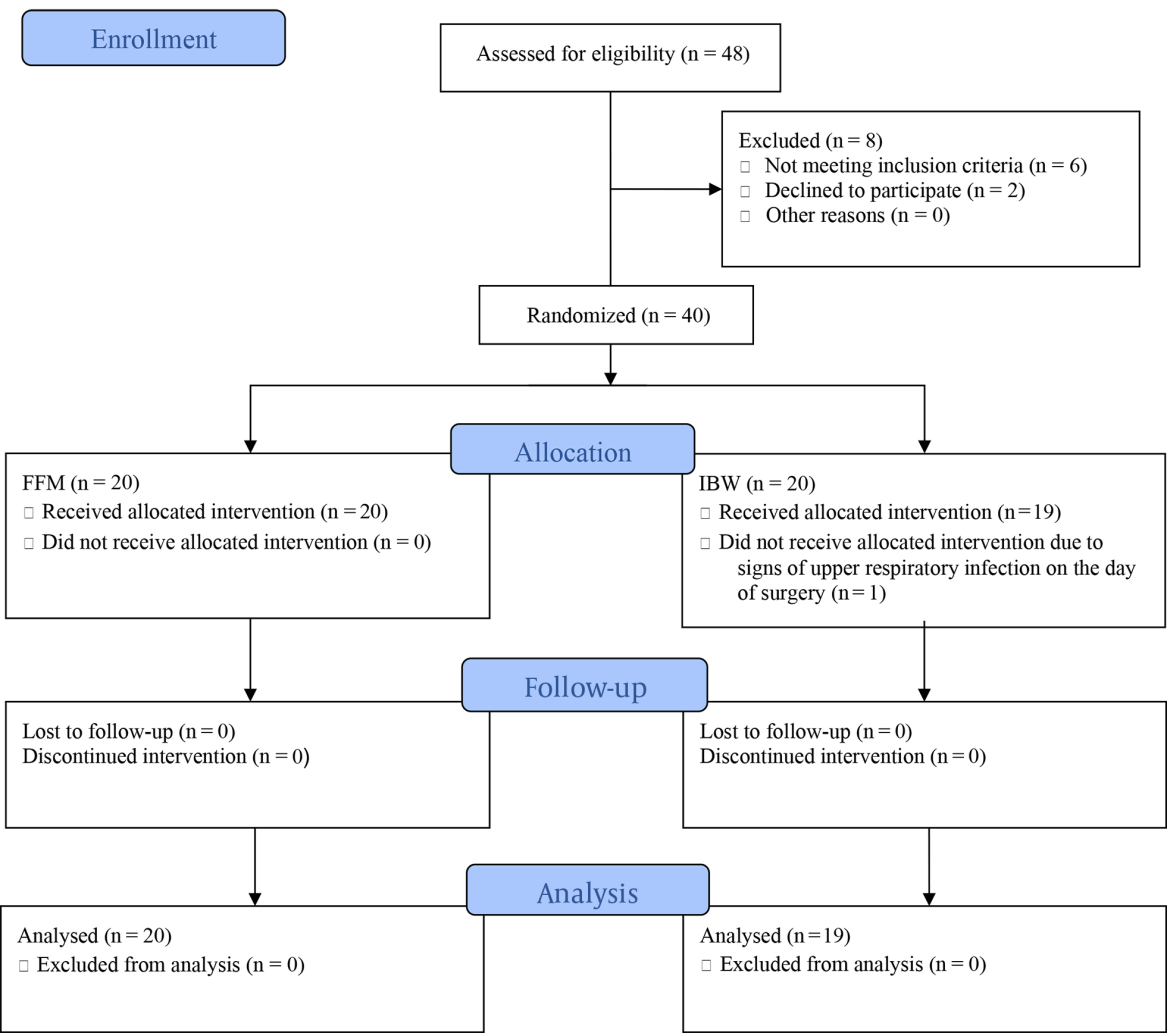
The consolidated standards of reporting trials (CONSORT) flow diagram of the present study

Figure 1 demonstrates the consolidated standards of reporting trials (CONSORT) flow diagram of this trial.

### 3.2. Patients Preparation

The day before surgery, the patient’s body weight (ie, TBW) and its FFM composition were determined using the bioelectrical impedance analysis (BIA; In-body 720, Biospace Korea). Ideal body weight was calculated using the Devine formula (27), ie, IBW (kg) = 50 kg (for men) or 45.5 kg (for women) + 0.9 kg per height in centimeters over 152.4 cm.

### 3.3. Anesthesia Protocol

Routine monitoring, including electrocardiography, non-invasive blood pressure monitoring, peripheral blood oxygen saturation and temperature monitoring, capnography, acceleromyography (TOF-Watch SX monitoring system, Organon, Ireland), and bispectral index (BIS) monitoring (BISPECTRAL VISTA monitoring system; Covidien Company, USA) were established by the time the patient entered the operating room. Two antecubital veins were cannulated using two 20-gauge catheters on both arms, and a bolus of 4 mL/kg of isotonic fluid was administered.

Patients were placed in a ramp position, with the ear canal aligned with the suprasternal notch to facilitate endotracheal intubation, and were pre-oxygenated for 3 min through a face mask, followed by an administration of fentanyl 2 µ/kg (LBW). After 2 min, propofol 1% (B. Braun, Germany) was administered for 1 min at a dose of 2 mg/kg, based on the study group weight. After the disappearance of the eyelash reflex, rocuronium (EMERSON, Netherlands) was administered to the other arm at a dose of 1 mg/kg based on LBW, and the trachea was intubated at TOF = 0. In case of inadequate anesthesia (ie, BIS > 60, straining during intubation, or eye-opening), an additional dose of propofol equal to 20% of the initial dose was administered. After intubation, propofol infusion was initiated, as maintenance of anesthesia, at a rate of 100 mcg/kg/h (TBW), and the infusion rate was adjusted to maintain a BIS between 40 and 60. Atropine was administered if the heart rate (HR) decreased below 50 BPM or if there was a more than 20% decrease from the baseline value. If blood pressure decreased by 40% or more of baseline blood pressure, vasopressors were administered.

### 3.4. Data Recording

The patient's mean arterial pressure (MAP) and HR were recorded 2 times, before the starting propofol administration and 2 min after that. The time from induction of anesthesia to loss of eyelash reflex and BIS ≤ 60 were recorded. The number of patients with inadequate anesthesia, requiring additional doses of propofol, was also recorded. Besides, the number of patients who required atropine or vasopressor administration was recorded. Propofol administration and data recording were accomplished by an anesthesiologist who was blinded to the study group. The dosing of propofol was 2 mg/kg, which was determined based on the weight information provided to the anesthesiologist.

### 3.5. Study Outcomes

The primary outcome was the time duration to reach BIS ≤ 60. Time to the disappearance of eyelash reflex, signs of inadequate anesthesia (ie, BIS > 60, straining during intubation, or eye-opening), requirements for additional doses, and hemodynamic indices (including HR and MAP at 2 times, before and 2 min after propofol induction dose) were also compared between the 2 groups.

### 3.6. Statistical Analysis

Data were analyzed using SPSS version 22.0 (Armonk, NY: IBM Corp). Continuous data with a normal distribution are presented as mean and SD. Data with skewed distributions are presented as medians and interquartile ranges. Categorical data are presented as counts and percentages. The Mann-Whitney U test or independent samples *t*-test was used to compare quantitative data between the 2 groups, the paired samples *t*-test was used to compare 2 quantitative data in each group, and the chi-square test or Fisher's exact test was used to compare qualitative data between the 2 groups. A sample size of 34 was estimated based on the study of Ingrande and Lemmens, with a power of 80% and a type I error rate (random error) of 0.05 (11). By taking into account an attrition rate of 20%, 40 patients were enrolled in the study.

## 4. Results

Of the 48 bariatric surgery candidates assessed for eligibility, 6 did not meet the inclusion criteria, and 2 declined to participate. The remaining 40 patients (20 in each group) were enrolled in the study and randomly assigned to the FFM or IBW groups. One patient was subsequently excluded from the IBW group due to exhibiting signs of upper respiratory tract infection on the day of surgery.

Patients in the 2 study groups were comparable in terms of age, gender ratio, BMI, FFM, and IBW (Table 1). The paired samples *t*-test showed that the mean values of measured FFM were higher than the mean values of calculated IBW in both FFM and IBW groups (P < 0.001 and P < 0.001, respectively). The study groups were also compared in terms of the percentage of patients with underlying conditions (Table 1; such that, 25% of the FFM group and 38.1% of the IBW group had underlying medical conditions, and the difference was not statistically significant (P = 0.368).

**Table 1.** Demographic Data ^a^

Variables	FFM Group (N = 20)	IBW Group (N = 19)	P-Value
**Age (y)**	41.7 ± 12.1	35.2 ± 11.5	0.086
**Gender (percentage of females)**	75.0	76.2	0.929
**BMI (kg/m** ^**2**^ **)**	44.1 ± 7.8	46.6 ± 5.1	0.238
**FFM (kg)**	64.2 ± 15.3	64.4 ± 10.4	0.964
**IBW (kg)**	57.42 ± 12.1	56.62 ± 8.2	0.812
**Underlying conditions (%)**	25	38.1	0.368

Abbreviations: BMI, body mass index; FFM, fat-free mass; IBW, ideal body weight.

^a^ Values are expressed as mean ± SD unless otherwise indicated.

As depicted in Table 2, regarding hemodynamic data, the mean values of MAP and HR before propofol administration (pre-intervention) were not significantly different between the study groups (P = 0.520 and P = 0.847, respectively). Post-intervention (2 min after propofol administration) mean values of MAP and HR were also similar in the FFM and IBW groups (P = 0.327 and P = 0.516, respectively). No vasopressor or atropine was required for patients in either study group.

**Table 2.** Hemodynamic Data ^a^

Variables	FFM Group (N = 20)	IBW Group (N = 19)	P-Value
**Pre- intervention MAP**	98.3 ± 14.3	95.6 ± 12.6	0.520
**Post-intervention MAP**	89.5 ± 15.9	84.0 ± 19.0	0.327
**Pre- intervention HR**	88.3 ± 10.5	89.1 ± 15.1	0.847
**Post-intervention HR**	83.7 ± 16.1	80.3 ± 16.7	0.516

Abbreviations: FFM, fat-free mass; IBW, ideal body weight; MAP, mean arterial pressure; HR, heart rate.

^a^ Values are expressed as mean ± SD.

The onset of action time of propofol is shown as “time to reach BIS ≤ 60” and “time to loss of eyelash reflex” in Table 3. The mean time to reach BIS ≤ 60 after propofol administration for induction of anesthesia in the FFM and IBW groups was 134.1 s (±47.6) and 148.7 s (±48.0), respectively. This difference was not statistically significant (P = 0.334). The mean time to loss of eyelash reflex after propofol administration was also similar in the FFM and IBW groups (76.5 ± 35.0 and 77.0 ± 18.0, respectively; P = 0.814).

**Table 3.** The Propofol Onset of Action ^a^

Variables	FFM Group (N = 20)	IBW Group (N = 19)	P-Value
**Time to reach BIS ≤ 60 (s)**	134.1 ± 47.6	148.7 ± 48.0	0.334
**Time to “loss of eyelash reflex” (s)**	76.5 ± 35.0	77.0 ± 18.0	0.814

Abbreviations: FFM, fat-free mass; IBW, ideal body weight; BIS, bispectral index.

^a^ Values are expressed as mean ± SD.

During the study, 2 patients in the FFM group and 8 patients in the IBW group showed signs of inadequate anesthesia and required additional administration of propofol. Fisher's exact test showed a significantly higher need for additional dose(s) in the IBW group (P = 0.032; Table 4). 

**Table 4.** Propofol Additional Dose Requirement ^a^

Additional Dose Required	FFM Group (N = 20)	IBW Group (N = 19)	Total	P-Value
**No**	18 (90.0)	11 (57.9)	29 (74.4)	0.032
**Yes**	2 (10.0)	8 (42.1)	10 (25.6)	

Abbreviations: FFM, fat-free mass; IBW, ideal body weight.

^a^ Values are expressed as No. (%) of the patients.

## 5. Discussion

This study was conducted to compare the dosing of propofol for induction of anesthesia based on FFM, measured with a BIA technique, with the dose based on IBW, calculated by using a formula based on height and gender of the patients in patients with MO.

In the current study, the BIS equal to or lower than 60 (BIS ≤ 60) and the disappearance of eyelash reflex were used to evaluate the onset of action of propofol. The Bispectral Index was taken as a reliable, objective measure; however, since there is a few seconds time lag for processing data, a clinical measure, the disappearance of eyelash reflex, was taken to estimate the propofol onset of action as well (28, 29). To evaluate the adequacy of anesthesia, episodes of BIS > 60 or straining during intubation or opening the eyes were determined. As shown in the results, the mean time to reach BIS ≤ 60 and disappearance of eyelash reflex were not statistically significant between the study groups. However, the need for additional doses during the time from finishing the propofol administration to tracheal intubation was significantly higher in the IBW group. That is, additional doses of propofol were required in 8 patients in the IBW group, while only 2 patients in the FFM group required additional doses. This suggests that FFM might be an appropriate body weight scalar for propofol administration in populations with obesity, and it may provide a more appropriate depth of anesthesia.

As previously mentioned, the altered PK-PD profile of the drug with MO and disproportionate increases in fat mass and LBW point to the need for precise dosing strategies in patients with MO (30, 31). Therefore, TBW-based dosing of propofol for induction of anesthesia might not be suitable for such patients. Wu et al. demonstrated that propofol EC_50_, the concentration required to produce half of the maximum effect, was significantly decreased in patients with MO. They divided the study participants into 3 groups: 1 control group and 2 study groups with MO (TBW and LBW). The patients in the control group and TBW group received propofol (2 mg/kg) based on TBW, and the patients in the LBW group received the propofol dose based on LBW. They found that propofol dosing based on TBW in patients with MO, compared to the control group and dosing based on LBW, provided a greater decrease in hemodynamic variables, including MAP, systolic and diastolic blood pressure, and cardiac output, measured at different time intervals from 0.5 to 20 min after propofol administration (23). Additionally, in the current study, no significant hemodynamic changes requiring intervention occurred at 2 min after propofol administration in participants of either study group. The reason for this could be that no dose, as large as a TBW-based dose, was administered to the patients of neither study group. Therefore, administering propofol for induction of anesthesia based on lower than TBW could decrease the risk of exaggerated hemodynamic alterations.

Some studies have evaluated LBW for propofol dosing for induction of anesthesia, and the findings are controversial. Ingrande et al. evaluated 60 morbidly obese patients (30 in each group; LBW or TBW) and 30 normal-weight controls (22). They administered propofol at a rate of 100 mcg/kg/h for induction of anesthesia based on the study group weight. Loss of consciousness was determined by dropping a weighted syringe. Their results showed similar doses required for anesthesia induction in the LBW and control groups. Patients in the TBW group received higher doses and, therefore, lost consciousness in less time. They suggested that LBW is a better weight scalar for propofol administration compared to TBW. Fat-free mass is made up of vital organs, bones, muscles, and extracellular fluid. Technically, there is a small difference between LBW and FFM, such that LBW further includes the lipids in cellular membranes. Therefore, the terms FFM and LBW can be used interchangeably when assessing body composition for drug delivery dosing (32, 33). From this perspective, it can be claimed that the results of the study of Ingrande et al. (22) are consistent with the results of the current study, suggesting that using FFM-based dosing of propofol for anesthetic induction, which aligns with a weight base similar to LBW, led to more favorable results. However, different criteria were used to evaluate loss of consciousness in the 2 studies. Fortunately, besides the subjective criterion (“loss of eyelash reflex”), the use of an objective criterion (“BIS”) in the current study might increase the accuracy of the results.

Subramani et al., in their study on patients with MO, administered a propofol anesthetic induction dose based on LBW in one group, while in the other group, the dose was based on the time it took for BIS to reach 50. Loss of consciousness was assessed using the responsive scores of the modified Observer’s Assessment Alertness/Sedation Scale (OAA/S) (34). They demonstrated that propofol dosing based on LBW did not provide adequate anesthesia compared to dosing based on a target endpoint of BIS of 50 in patients with MO, and additional doses were required. Although the criteria for evaluating the depth of anesthesia was different in the current study, and the BIS value was evaluated in all the patients as one of the criteria for assessing loss of consciousness, the results of the current study are inconsistent with the study of Subramani et al. (34) in that propofol anesthetic induction dose based on FFM, a similar weight base to LBW, provided adequate anesthesia in the current study, and extra doses of propofol were required in only 10% of patients in this study group.

In the current study, the time to reach BIS ≤ 60, the disappearance of eyelash reflex, or the hemodynamic indices examined in the 2 groups were not significantly different. However, the need for additional doses of propofol due to inadequate anesthesia was higher in the IBW group, favoring the appropriateness of FFM-based dosing of propofol as a choice for induction dose.

We believe the strength of the current study is the individualization of the study weight for each patient in one of the study groups. In the studies mentioned above, LBW was derived from TBW by calculating a formula (22-24). Ideal body weight in the current study was also derived from a formula calculating the height and sex of the patient. It seems that using a formula could not account for the alterations in the proportion of body composition of the patients. However, FFM, the other weight scalar studied in this clinical trial, was individualized to each patient using the BIA method. As a matter of fact, the appropriateness of FFM-based dosing of propofol dose for induction of anesthesia compared to IBW-based dosing, based on the results of the current study, cannot be solely attributed to the slightly higher mean value of FFM compared to the mean value of IBW; however, the individualized-based dosing of FFM might have made an important role in providing more favorable result with propofol dosing based on FFM. Although BIA may overestimate FFM, using multi-frequency BIA is shown to reduce this bias (35). It seems that measuring FFM based on each individual patient resulted in less requirement of additional propofol doses in this study.

Last but not least, given the similarity between FFM and LBW with a slight difference and the impracticability of routinely measuring FFM for all patients in all medical centers, we also recommend the administration of propofol based on LBW as a practical approach in light of the findings of this study.

### 5.1. Conclusions

Propofol dosing based on the weight of FFM provides a more favorable depth of anesthesia compared to dosing based on IBW for induction of anesthesia in patients with MO.

## Data Availability

The dataset presented in the study is available on request from the corresponding author during submission.

## Clinical Trial Registration Code:

IRCT20201024049135N1.
